# Heinrich Sachs (1863–1928)

**DOI:** 10.1007/s00415-014-7517-2

**Published:** 2014-10-26

**Authors:** Stephanie J. Forkel

**Affiliations:** 1 Division of Psychology and Language Sciences, Research Department of Clinical, Educational, and Health Psychology, University College London, London, UK; 2 Natbrainlab, Department of Neuroimaging, Institute of Psychiatry, Psychology and Neuroscience, King’s College London, London, UK

The nineteenth century witnessed some of the greatest neuroanatomists of all times. Amongst them is the largely forgotten Heinrich Sachs, a student of Carl Wernicke in Breslau.

Sachs was a German neurologist, born in 1863 in Halberstadt a town in the German state of Saxony-Anhalt and the capital of the district of Harz. Despite his upbringing in a lower-income background, he was able to study medicine in Berlin where he graduated in 1885 with his doctoral thesis on amyotrophic lateral sclerosis. Thereafter, Sachs practised as a physician for some years before starting his specialisation within Carl Wernicke’s laboratory at the University Hospital of Breslau [1]. Sachs habilitated in 1897 in Psychiatry and Neurology with his work on the accruement of spatial perception through sensory impressions [2].

**Fig. 1:**
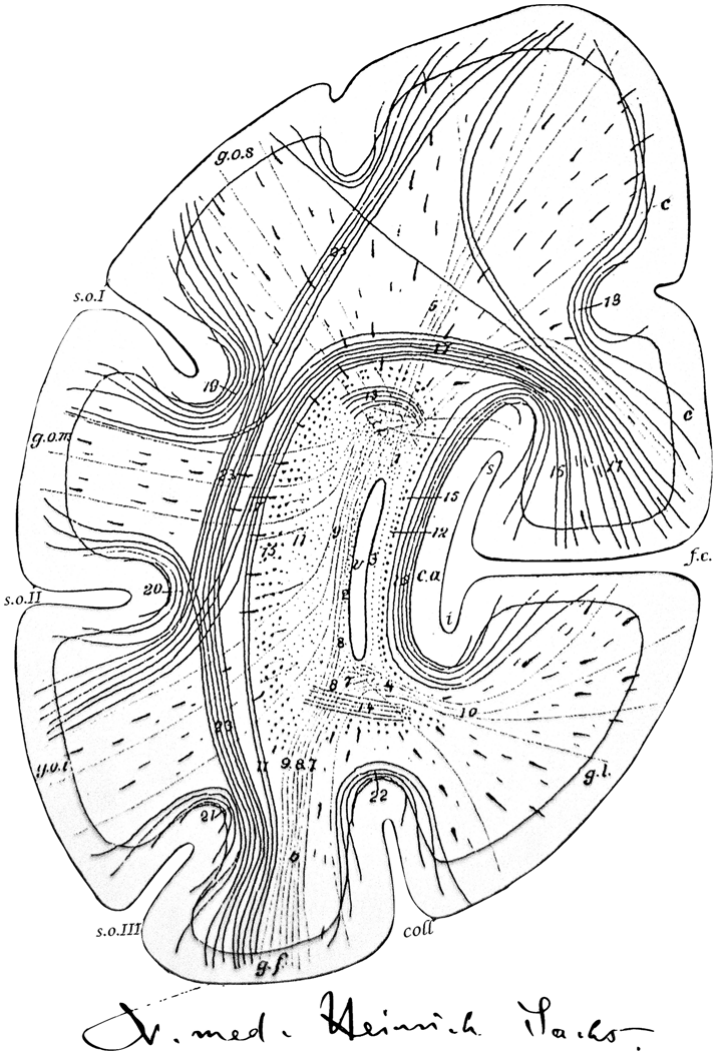
Sachs’ signature as shown in his private files from 1897 and a frontal section through the right occipital lobe shown as schematic representation of the white matter arrangement (taken from his atlas [4, 5]). Anatomical labels identify the following structures: *v* ventricle, *f.c.* fissura calcarina (calcarine fissure), *coll.* collateral sulcus, *s.o.I* sulcus occipitalis superior, *s.o.II* sulcus occipitalis medium, *s.o.III* sulcus occipitalis inferior, *g.l.* gyrus lingualis, *g.f.* gyrus fusiformis, *g.o.s.* superior occipital gyrus, *g.o.m.* middle occipital gyrus, *1–10* forceps, *11–14* stratum sagittale internum, *15* stratum sagittale externum, *16* stratum calcarinum, *17* stratum cunei transversum, *18* stratum proprium cunei, *19* stratum proprium s.o.I, *20* stratum proprium s.o.II, *21* stratum proprium s.o.III, *22* stratum proprium coll., *23* stratum profundum convexitatis

The most comprehensive information accessible today originates from Sachs’ personal files, which contain his signature (Fig. 1). These files enclose a reply to the demand of the principal of the royal university acting upon order of the royal ministry, which insisted that professors of their universities disclose their personal and professional relations. In this document, Sachs stated that his confession was Jewish-protestant and as a remark added that he was also working as specialist registrar at the hospital for accidents and emergencies. This position was to influence one of his last publications on traumatic neurosis [3].

Sachs was married with one child. His wife was brought up within an established merchant family in Breslau and their daughter was the poet Lessie Sachs (1897–1942) who emigrated to the US during the Nazi era.

In 1892 Sachs published the first part of a white matter atlas, which was dedicated to the investigation of the anatomy of the human brain. This endeavour was devoted to the study of the connectional anatomy of the occipital lobe and the adjacent temporal and parietal lobes [4]. Carl Wernicke expressed his deep appreciation and enthusiasm for this work within the preface. This wonderful elaborate effort demonstrated white matter connections within the occipital lobe (intralobar U-shaped fibres) in parallel to trajectories reaching adjacent cortices (interlobar association pathways). Sachs highlighted the importance of studying anatomy in the healthy brain prior to examining pathological, embryological or animal specimens. He further claimed that to establish a trajectory of a neuronal pathway a minimum of two methods was required to partially overcome the shortcomings inherent to each method [4]. His remarkable atlas has recently been translated [5] and replicated using the Klingler method [6].

Of note, it was also within this work that Sachs argued that the origin of the “superior fronto-occipital fasciculus” derived from callosal fibres, which were unable to transverse the hemispheric midline [7, 8]. Owing to this observation, it was recently suggested that the “Probst bundle” (the alternative name used for the superior fronto-occipital fasciculus) be renamed as the “Sachs-Probst bundle” [7]. Sachs was, however, not the first to publish this observation [8]. His name has been adopted for other pathways and structures in the brain including the fasciculus occipitalis transversus of Vialet and Sachs [6].

Beyond his detailed anatomical interest, Sachs was captivated by the structural- functional relationships in the brain and attempted to use his anatomical knowledge to explain behaviour. Being a neurologist and a student of Wernicke, he repeatedly scrutinised anatomical models of aphasia [9, 10]. Sachs sternly critiqued the schematic models as oversimplified and highlighted the importance of the white matter connections between cortical regions: “The doctrine presented here had already been subjected to substantial criticism by Freud in 1892. Nevertheless due to its convenience, simplicity and apparent tangibility it withstood the critique. […] It should be highlighted that for Wernicke the content centre [Begriffszentrum] has never been anything else but the entire cortex. His choice of the term “transcortical” does not reflect an entity outside the brain, such as the soul, but was dedicated to the fibre pathways which are interconnecting cortical areas”. Of contemporary interest is his closing paragraph [9]: “The question of utmost interest is whether the right hemisphere, which is identical to the left in its anatomy, can functionally compensate when the language centres, and in particular the sensory language centre [superior temporal gyrus], in the left hemisphere are damaged. This compensation is possible and does occur if an illness affects a child within its first years of life”.

In his habilitation on the accruement of spatial perception Sachs dichotomised all sensory impressions into simple and complex inputs. The first group consists of taste, olfaction and thermoception. These perceptions can occur in different qualities with altered intensities and elicit distinct feelings; yet, no spatial or temporal relations are experienced or encoded in memory. In contrast, auditory, visual, and haptic inputs require relations. For example, auditory input necessitates the recognition of tones (simple input) in parallel with the relation between them, i.e. harmonies and melodies (complex input). Sachs believed that simple perception is innate whereas complex perception is acquired and thus a higher brain function which reaches beyond passive sensations.

For Heinrich Sachs, the brain was the master and the body was the emissary: “The function of the nervous system is to orchestrate the functions of all individual organs […]. This unique purpose of the nervous system to coordinate all other parts of the body to produce a unified action is represented in its form. It is made of nothing but a net of fibres. At times these fibres are propagating to the body and are in contact with all other organs. Other fibres combine as big tangles to form nerve centres in which the regulation of the bodily functions is performed”.

Sachs died aged 65 years in Breslau in 1928. The circumstances and causes of his death are not currently known.
